# Divergent Hepatic Outcomes of Chronic Ketone Supplementation: Ketone Salts Preserve Liver Health While Ketone Esters and Precursors Drive Inflammation and Steatosis

**DOI:** 10.3390/ph18101436

**Published:** 2025-09-25

**Authors:** Csilla Ari, Dominic P. D’Agostino

**Affiliations:** 1 Behavioral Neuroscience Research Laboratory, Department of Psychology, University of South Florida, Tampa, FL 33620, USA; 2 Ketone Technologies LLC, Tampa, FL 33612, USA; ddagosti@usf.edu; 3 Laboratory of Metabolic Medicine, Department of Molecular Pharmacology and Physiology, University of South Florida, Tampa, FL 33612, USA; 4 Institute for Human and Machine Cognition, Ocala, FL 34471, USA

**Keywords:** exogenous ketones, liver histopathology, TNF-α, arginase, globulin, creatinine, ketone supplementation, safety

## Abstract

**Background/Objectives:** Exogenous ketone supplements elevate circulating ketones without carbohydrate restriction, but their long-term hepatic safety remains unclear. This study evaluated the formulation-dependent impact of chronic ketone supplementation on liver histopathology, inflammatory signaling, and systemic biomarkers in rats. **Methods:** Male Sprague-Dawley rats were orally administered 1,3-butanediol (BD), medium-chain triglycerides (MCTs), ketone ester (KE), ketone electrolytes/salts (KSs), or a ketone salt–MCT combination (KSMCT) for 4 weeks. In a separate arm, animals received standard diet (SD), or SD supplemented with low-dose KE (LKE) or high-dose KE (HKE), for 83 days. Liver structure was assessed by hematoxylin and eosin staining with quantification of red blood cell density and lipid accumulation. Inflammatory and metabolic responses were evaluated by TNF-α and arginase immunohistochemistry. Serum biochemistry included glucose, proteins, electrolytes, and liver and kidney function markers. **Results:** BD and KE induced macrovesicular steatosis, vascular congestion, and elevated TNF-α and arginase expression, consistent with hepatic stress. MCT caused moderate hepatocellular ballooning and lipid deposition, whereas KS preserved near-normal hepatic morphology. KSMCT produced intermediate effects, reducing lipid accumulation and TNF-α compared with MCT or KE alone. KE supplementation caused dose-dependent reductions in globulin and elevations in creatinine, while HKE reduced sodium and glucose levels. **Conclusions:** Chronic hepatic responses to exogenous ketones are highly formulation dependent. KS demonstrated the most favorable safety profile under the tested conditions, maintaining normal hepatic structure, while BD and KE elicited adverse changes. Formulation choice is critical for the safe long-term use of exogenous ketones.

## 1. Introduction

Exogenous ketone supplementation has emerged as a novel nutritional intervention capable of rapidly elevating circulating levels of beta-hydroxybutyrate (BHB), the primary ketone body produced during carbohydrate restriction, fasting, or ketogenic dieting [1]. The capacity to induce a state of nutritional ketosis independent of dietary adherence holds significant therapeutic promise for metabolic disorders, neurological diseases, and performance optimization [1,2,3].

Several forms of exogenous ketones are available, including ketone electrolytes/salts (KSs, typically racemic mixtures of D(R)- and L(S)-BHB bound to minerals), ketone esters (KEs, such as (R)-3-hydroxybutyl (R)-3-hydroxybutyrate), and ketogenic precursors like 1,3-butanediol (BD) and medium chain triglycerides (MCTs). These compounds differ in their pharmacokinetics, tolerability, and metabolic effects. The D-isomer of BHB, the only isomer that can be measured by handheld meters, is preferentially oxidized for energy by peripheral tissues, while the L-isomer is metabolized more slowly and may exert regulatory effects on inflammation and gene expression [4,5]. The bioactivity of L-BHB is less well characterized, but it is believed to contribute to systemic responses distinct from energy production [5].

Emerging studies have explored the physiological impact of exogenous ketones on various blood parameters, particularly those associated with metabolic function [6,7]. In our earlier studies, administration of ketone electrolyte salts and esters to Sprague-Dawley rats resulted in significant elevations in blood BHB without adverse effects on glucose homeostasis [8,9,10]. However, variations in responses were observed depending on formulation and dose. Myette-Côté et al. (2019) reported that KE ingestion in humans blunted postprandial glycemia and lipolysis, suggesting a potential regulatory role in energy metabolism [7]. Our previous studies included behavioral responses after chronic administration of exogenous ketone supplementation, showing improved motor performance, anxiolytic effect, in addition to neuroprotective effects [8,9,10]. Despite these promising results, long-term safety data remain limited, especially regarding potential effects on hepatic and renal function. Parameters such as TNF-α, arginase, globulin, creatinine and liver enzymes provide insight into systemic impacts of ketone supplementation, but are not reported in the current literature. As exogenous ketones become increasingly popular among athletes, patients with chronic diseases, and health enthusiasts, a deeper understanding of their influence on these biomarkers is crucial.

Tumor necrosis factor-alpha (TNF-α) is a key pro-inflammatory cytokine elevated during hepatic stress and injury [11]. In the liver, high TNF-α levels are linked to conditions, such as NAFLD, alcoholic hepatitis, and drug-induced injury [12]. By driving hepatocellular injury, fibrogenesis, and immune cell recruitment, TNF-α serves as a critical marker of inflammation. Measuring its expression helps assess how different ketone formulations influence hepatic immune responses.

Arginase, a urea cycle enzyme in the liver, converts arginine to urea and ornithine [13]. Beyond nitrogen disposal, it also modulates immune responses and tissue repair. Elevated activity is linked to liver injury, fibrosis, and oxidative stress [14], and its expression rises with inflammation and metabolic load, making it a sensitive biomarker of hepatic adaptation or dysfunction.

Hepatic integrity was evaluated by hematoxylin and eosin (H&E) staining to assess structural changes, lipid accumulation, and vascular congestion induced by different ketone formulations. Red blood cell (RBC) density within hepatic sinusoids was measured as an indicator of microvascular congestion and altered hepatic blood flow, which may reflect systemic stress and long-term safety concerns [15]. Lipid accumulation was also quantified, since prolonged ketone supplementation can disrupt the balance between fatty acid uptake, storage, and oxidation. Excessive lipid deposition (macro- or microvesicular steatosis) may indicate maladaptation and risk of steatohepatitis [16], while reduced lipid droplets suggest improved metabolic flexibility and enhanced oxidation [17].

Evaluating TNF-α levels through immunohistochemistry enables assessment of the inflammatory response to exogenous ketone supplementation [18]. Likewise, histological evaluation of the liver, including arginase expression, allows insight into metabolic adaptations or hepatic burden following prolonged ketone supplementation intake [19,20,21,22,23].

Globulin, produced in the liver, supports immune function through antibody production, carrier activity, and inflammation regulation. Reduced globulin may signal hepatic insufficiency, malnutrition, or immune suppression [24]. Creatinine is a key renal marker, with elevations indicating impaired filtration, protein catabolism, or altered hydration [25]. Changes in these parameters can reveal subtle hepatic or renal adaptations to chronic ketone exposure.

Other routinely assessed markers, such as albumin, glucose, and liver enzymes (ALT, ALP), help to delineate the broader metabolic and physiological landscape influenced by exogenous ketones. Glucose and insulin responses to ketones have been studied extensively in acute contexts, but chronic effects on hepatic biosynthesis, protein metabolism, and renal clearance remain poorly defined [26,27].

To address this gap, the present study also evaluates a range of blood chemistry markers in Sprague-Dawley rats after chronically administering either standard diet (SD), or SD supplemented with low-dose ketone ester (LKE), or high-dose ketone ester (HKE). The aim is to determine whether ketone supplementation induces significant changes in liver and kidney biomarkers, lipid metabolism, or blood biochemistry. We hypothesize that KE consumption may alter blood biochemistry levels in a dose-dependent manner, potentially reflecting systemic adaptation to ketone metabolism.

Recent investigations highlight potential dose-dependent renal stress markers, including elevated creatinine, as well as changes in liver protein synthesis and globulin concentrations during prolonged ketone ester administration [19,25]. Furthermore, enantiomer-specific analyses suggest that L-BHB may persist longer in circulation and confer anti-inflammatory and cardioprotective benefits distinct from D-BHB [5,15]. These findings underscore the importance of evaluating different formulations, dosing, and isomer composition for long-term supplementation.

Despite promising therapeutic potential, long-term preclinical and translational data remain limited, particularly with respect to liver histology, inflammatory biomarkers, and renal physiology. The present study aimed to address this gap by systematically evaluating hepatic and renal function, as well as core blood parameters relevant to clinical and translational health applications, systemic responses to chronic administration of diverse ketone formulations in rats, integrating histological, immunohistochemical, and biochemical analyses to help assess safety and tolerability.

## 2. Results

### 2.1. Histological Analysis Revealed Formulation-Specific Effects on Liver Structure and Markers of Hepatic Inflammation

**Figure 1:**
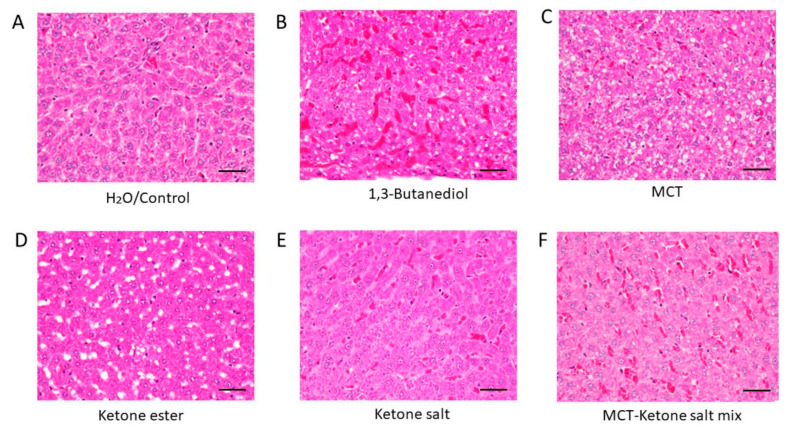
H&E-stained liver sections showing tissue morphology across treatment groups after 4 weeks of chronic administration (40×). (**A**) H_2_O/Control: Normal liver histology with intact architecture and minimal RBCs. (**B**) 1,3-Butanediol: Pronounced steatosis, vascular congestion, and dense RBC infiltration. (**C**) MCT: Hepatocellular ballooning, small fat vacuoles. (**D**) Ketone Ester: Liver steatosis. (**E**) Ketone Electrolyte/Salt: Near-normal liver morphology, well-preserved tissue with low RBC density. (**F**) Ketone Salt–MCT mix: Modest vascular congestion and increased RBC density. RBC: red blood cell. Scale bar: 50 µm.

**Figure 2:**
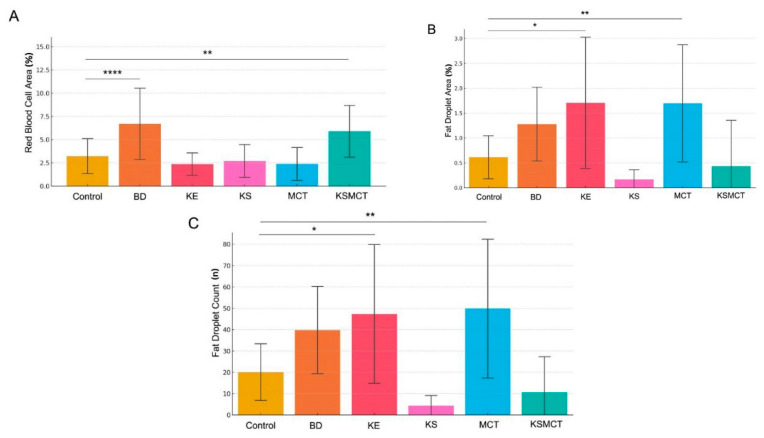
Comparison of red blood cell (RBC) area (**A**), fat droplet area (**B**) and fat droplet count (**C**) in the liver across treatment groups after 4 weeks of chronic administration. (**A**) Mean RBC area per histological section was quantified for six treatment groups. Treatment with BD significantly increased the RBC area, compared to all other groups (*p* < 0.0001), while the KSMCT group also showed a modest, but significant, elevation compared to Control (*p* = 0.0071), KE (*p* < 0.0001), KS (*p* = 0.0006), and MCT (*p* < 0.0001). KE, KS, and MCT alone did not differ significantly from Control or each other. (**B**) Fat droplet area quantification across treatment groups highlights divergent effects of ketone formulations on hepatic lipid deposition. Mean fat droplet area per image was quantified in liver histological sections from six experimental groups: Groups KE and MCT exhibited significantly greater mean fat droplet areas, compared to Control (*p* < 0.05 and *p* < 0.01, respectively). KE-treated samples also showed significantly higher fat area than KS (*p* < 0.0001) and KSMCT (*p* < 0.01). Conversely, KS exhibited significantly lower fat area, compared to MCT (*p* < 0.0001). MCT samples had greater fat accumulation than KSMCT (*p* < 0.001). (**C**) Fat droplet counts across experimental groups, suggesting treatment-specific effects on lipid accumulation. Bar graphs show the mean number of fat droplets per image across six treatment groups: Notably, KE and MCT groups exhibited significantly higher fat droplet counts, compared to Control and KS, while KS had the lowest counts overall. Control, BD (1,3-Butanediol), KE (Ketone Ester), KS (Ketone Electrolyte/Salt), MCT (Medium-Chain Triglyceride), and KSMCT (Ketone Salt + MCT). **** *p* < 0.0001, ** *p* < 0.01, * *p*  <  0.05.

Hematoxylin and eosin (H&E)-stained liver tissue revealed distinct morphological changes across treatment groups (Figure 1 and Figure 2, Table 1). Control (H_2_O) animals exhibited normal liver architecture with intact hepatocyte structure, regular sinusoidal spacing, and sparse RBC presence. The ketone electrolyte/salt group (KS) displayed similarly preserved hepatic histology, suggesting minimal impact from this formulation, with only mild RBC presence. The KSMCT treatment resulted in increased RBCs within sinusoids, and mild sinusoidal dilation, indicating vascular congestion. MCT alone induced hepatocellular ballooning, frequent lipid vacuoles, reflecting moderate hepatic stress. The KE group showed macrovesicular fat accumulation, consistent with moderate steatosis. The BD group exhibited the most severe alterations, including widespread fat deposits, hepatocyte swelling, dense RBC accumulation, and sinusoidal congestion, characteristic of pronounced steatosis, hepatic stress, and potential toxicity.

### 2.2. Red Blood Cell Area Is Significantly Elevated in 1,3-Butanediol (BD) and KSMCT Groups

Quantitative analysis of RBC area across treatment groups revealed significant differences following 4 weeks of chronic administration (Figure 1 and Figure 2A, Table 1). In the BD group, there was markedly increased RBC area, compared to all other groups (all *p* < 0.0001), including Control, KE, KS, MCT, and KSMCT. Additionally, the KSMCT group exhibited significantly greater RBC area, compared to Control (*p* = 0.0071), KE (*p* < 0.0001), KS (*p* = 0.0006), and MCT (*p* < 0.0001). In contrast, no significant differences were observed between the Control, KE, KS, and MCT groups, indicating that these treatments did not independently alter RBC area. These results show that BD, and to a lesser extent the KSMCT, significantly influence RBC morphology or density in liver tissue.

### 2.3. Lipid Accumulation Significantly Increased by KE and MCT Treatments

Quantification of fat droplet area in liver sections revealed significant differences among treatment groups (Figure 1 and Figure 2B). Both the KE and MCT groups displayed significantly increased mean fat droplet area, compared to the Control group (*p* < 0.05 and *p* < 0.01, respectively). KE also exhibited a markedly greater fat area than both the KS (*p* < 0.0001) and the KSMCT group (*p* < 0.01). Additionally, the fat droplet area in the MCT group was significantly higher than in the KS group (*p* < 0.0001) and in the KSMCT group (*p* < 0.001). No significant differences were observed between Control, KS, or KSMCT, and BD groups, while there was a trend of increased droplet area in BD.

In the analysis of fat droplet count across treatment groups, significant differences were observed between several experimental conditions (Figure 1 and Figure 2C). Both the KE and MCT groups exhibited markedly higher fat droplet counts, compared to the Control group (*p*  =  0.0152 and *p*  =  0.003, respectively). The KS group had the lowest droplet counts overall, showing highly significant reduction when compared to BD, KE, and MCT groups (all *p*  <  0.0001). The KSMCT group demonstrated intermediate values that were significantly lower than KE (*p*  =  0.0002) and MCT (*p*  <  0.0001), but not significantly different from Control, KS or BD. These findings highlight distinct effects of each treatment on hepatic lipid accumulation, with KS consistently associated with reduced fat droplet presence.

### 2.4. Increased TNF-α Levels in BD, KE and MCT Groups, While Decreased Levels in KS and KSMCT Groups

**Figure 3:**
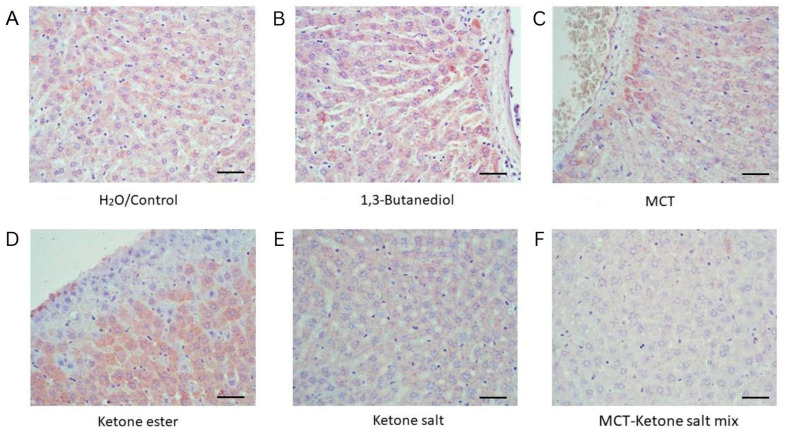
Representative liver sections stained for TNF-α across six treatment groups after 4 weeks of chronic administration (40×). (**A**) H_2_O/Control: Minimal TNF-α staining, representing baseline inflammation. (**B**) 1,3-Butanediol: Strong staining, indicating significant hepatic inflammation. (**C**) MCT: Moderate TNF-α expression. (**D**) Ketone Ester: Marked peri-central inflammation. (**E**) Ketone Electrolyte/Salt: Low TNF-α reactivity, similar to or lower than control. (**F**) Ketone Salt + MCT mix: Mild staining, low TNF-α reactivity. Scale bar: 50 µm.

**Figure 4:**
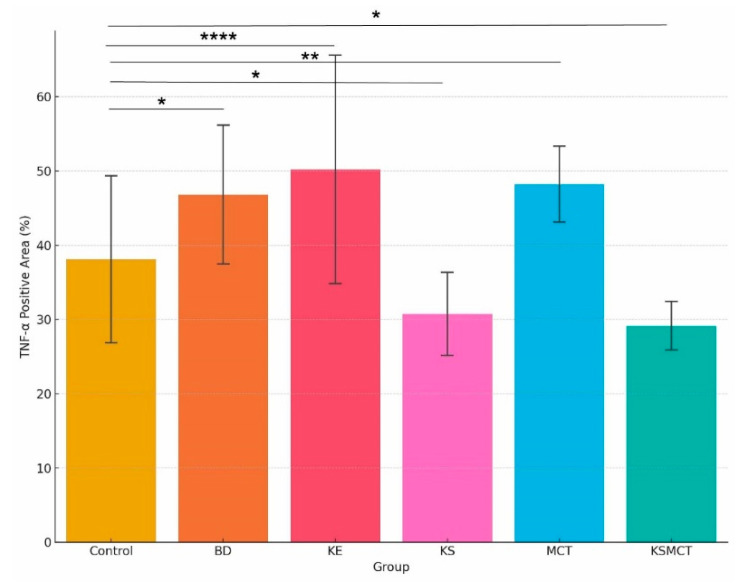
Quantification of TNF-α positive areas across experimental groups after 4 weeks of chronic treatment. Bar graph showing the percentage of tissue area positively stained for TNF-α in each treatment group: BD (*p* = 0.011), KE (*p* < 0.0001) and MCT (*p* = 0.0066) groups showed significantly more TNF-α positive areas, while KS (*p* = 0.044) and KSMCT (*p* = 0.0334) groups showed significantly less TNF-α positive areas, compared to control. Control, BD (1,3-butanediol), KE (ketone ester), KS (ketone electrolyte/salt), MCT (medium-chain triglyceride), KSMCT (ketone salt + MCT). * *p*  <  0.05, ** *p * <  0.01, **** *p*  <  0.0001.

Histological examination of liver tissue stained for TNF-α revealed differential inflammatory responses across the six treatment conditions (Figure 3 and Figure 4, Table 2). The Control group exhibited a mean TNF-α positive area of 36.1%, which was significantly lower than the levels observed in the BD (43.5%, *p* = 0.011), KE (48.2%, *p* < 0.0001), and MCT (46.2%, *p* = 0.0066) groups. In contrast, the KS (28.4%) and KSMCT (27.2%) groups displayed a significantly reduced TNF-α positive area, compared to Control (*p* = 0.044 and *p* = 0.0334, respectively). These results indicate a marked increase in TNF-α expression in response to BD, KE and MCT supplementation, and a suppressive effect in KS and KSMCT groups.

### 2.5. Elevated Arginase Level in BD and KE Groups

**Figure 5:**
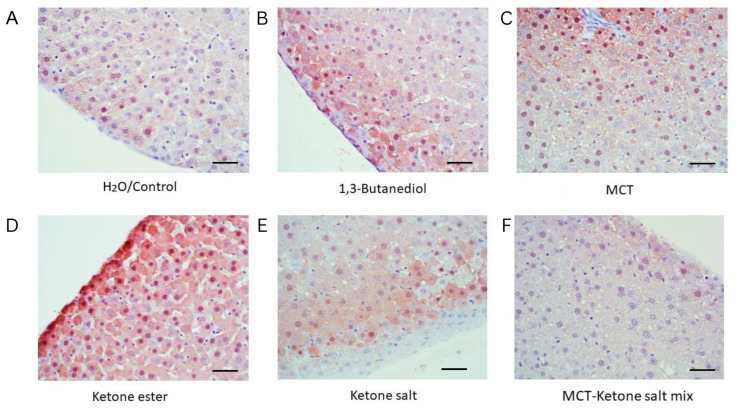
Representative liver sections stained for arginase expression across treatment groups after 4 weeks of chronic administration (40×). (**A**) H_2_O/Control: Low baseline arginase expression. (**B**) 1,3-Butanediol: Elevated arginase signal, likely reflecting enhanced metabolic demand. (**C**) MCT: Moderate arginase staining, reflecting adaptive hepatic metabolism. (**D**) Ketone Ester: High expression, indicating increased metabolic or oxidative stress. (**E**) Ketone Electrolyte/Salt: Low arginase signal, comparable to control. (**F**) Ketone Salt + MCT mix: Low arginase signal. Scale bar: 50 µm.

**Figure 6:**
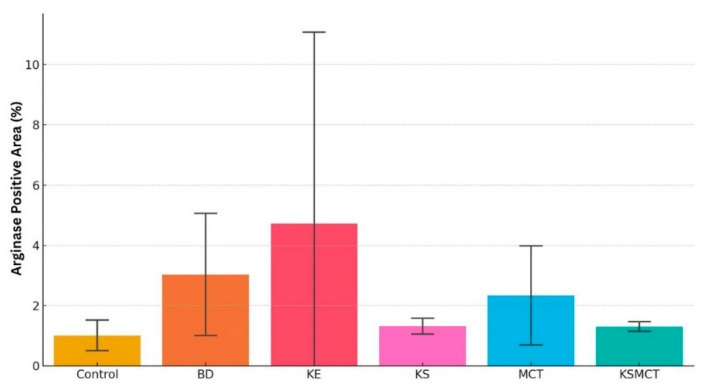
Comparison of arginase expression as a marker of anti-inflammatory response across treatment groups. Bar graphs representing arginase-positive area (%) in tissue sections from six groups. There was a trend of elevated arginase levels in BD and KE groups and a mild elevation in MCT group, while the arginase level was comparable to control in KS and KSMCT groups. Control, BD (1,3-butanediol), KE (ketone ester), KS (ketone electrolyte/salt), MCT (medium-chain triglycerides), and KSMCT (combined ketone salt and MCT).

Arginase staining in liver tissue sections also revealed formulation-specific responses (Figure 5 and Figure 6, Table 3). Control animals displayed low levels of arginase expression, consistent with physiological baseline. Both the BD and KE groups exhibited a trend of markedly increased arginase staining, indicating elevated hepatic metabolic activity or stress adaptation. The MCT group showed a trend of moderate arginase levels, suggesting an intermediate metabolic response. In contrast, KS-treated animals had minimal arginase staining, resembling the control group, while the KSMCT group exhibited mild expression, slightly above control.

### 2.6. Blood Chemistry Analysis Revealed Significant Group-Level Differences in Key Biomarkers

**Figure 7:**
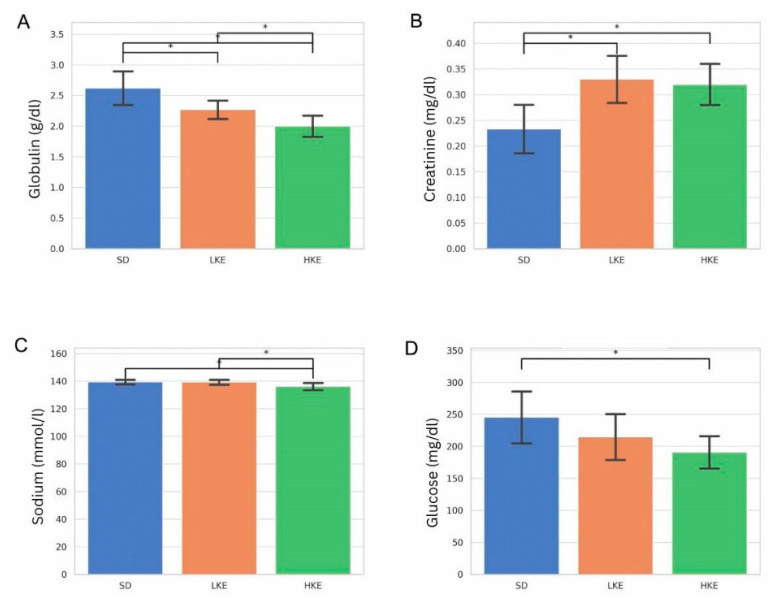
Blood chemistry analysis revealed significant differences in key biomarkers in response to chronic KE supplementation. (**A**) Globulin levels by treatment group. There was a statistically significant decrease in LKE and HKE groups, compared to control. LKE group had significantly higher globulin level than HKE. (**B**) Creatinine levels by treatment group. Creatinine value showed a significant lower level in the SD group, compared to LKE (*p* = 0.0005) and HKE (*p* = 0.0009). No difference was observed between LKE and HKE groups. (**C**) Sodium levels by treatment group. Serum sodium level was significantly lower in HKE group, compared to control and LKE. (**D**) Blood glucose levels by treatment group. Blood glucose concentration was significantly lower in HKE group, compared to control. SD (standard diet), LKE (low-dose ketone ester), and HKE (high-dose ketone ester) groups. * *p* < 0.05.

Significant differences were observed in several blood biomarkers among the three treatment groups: standard diet (SD), low-dose ketone ester (LKE), and high-dose ketone ester (HKE) (Figure 7) in response to chronic treatment.

Globulin levels were significantly lower in the HKE group, compared to the SD group (*p* = 0.000093), suggesting potential effects on hepatic protein synthesis or immune function (Figure 7A). Creatinine levels were significantly higher in both the LKE and HKE groups, compared to SD (*p* = 0.000534 and *p* = 0.000933, respectively), indicating a potential renal response to chronic KE supplementation (Figure 7B).

Sodium and glucose levels showed decrease in HKE group, compared to control (*p* = 0.005659 and *p* = 0.011096, respectively, Figure 7C,D).

Other markers, including BUN, ALT, ALP, albumin, potassium, chloride and bilirubin, did not differ significantly between groups (Supplementary Table S1). ALT levels in SD controls were highly variable, often above the expected reference range, whereas both LKE and HKE groups displayed ALT values closer to normal, suggesting that KE supplementation did not exacerbate hepatocellular enzyme leakage. ALP values for all groups remained within the physiological interval, with slightly higher means in SD compared to KE groups. Total bilirubin concentrations remained stable across all conditions and within reference limits. Together, these results demonstrate that routine blood chemistry markers did not indicate liver injury in response to chronic KE supplementation, underscoring that histological and molecular endpoints provide more sensitive indicators of hepatic stress.

All animals remained in healthy weight range for their age even though the rate of weight gain changed with ketone supplementation, as previously reported [6,9]. D-BHB levels were as previously described [6,9].

## 3. Discussion

The findings from this study contribute to a growing body of literature on the histological and blood biomarker effects of exogenous ketone supplementation. The observations suggest a dose- and formulation-dependent impact of exogenous ketones on liver structure and vasculature, with ketone electrolytes/salts (KSs) demonstrating the most favorable histological profile after chronic treatment under the experimental conditions tested. The results further support the notion that chronic use of D,L-BHB ketone electrolytes/salts elicit the least change in the histology of the liver, hepatic enzyme induction and may represent the most favorable formulation for long-term use.

Taken together, systematic evaluation of RBC density and lipid accumulation provides a comprehensive view of hepatic adaptation to chronic ketone administration. These histological endpoints serve as early markers of both beneficial and adverse outcomes, offering critical translational relevance in assessing whether different ketone formulations support liver health, mitigate lipid burden, or predispose to microvascular and metabolic stress. This integrated histological approach complements biochemical measurements and strengthens the overall assessment of safety and efficacy in chronic ketone supplementation studies.

The observed increase in RBC area in the BD and KSMCT treatment groups suggests a potential effect of ketone-based interventions on hepatic microvascular dynamics (sinusoidal dilation) or erythrocyte infiltration in the liver. The substantial elevation in RBC area induced by BD may reflect increased hepatic perfusion or altered endothelial permeability, possibly due to BD’s rapid conversion to β-hydroxybutyrate due to alcohol dehydrogenase (and aldehyde dehydrogenase) and its systemic metabolic effects. Similarly, the moderate yet significant increase in RBC area in the KSMCT group suggests that the combined effects of exogenous ketones and medium-chain triglycerides may synergistically influence vascular tone or RBC trafficking within the hepatic tissue. Notably, neither KE, KS, nor MCT alone produced significant changes in RBC area, compared to control, indicating that the effects observed with BD and KSMCT may rely on unique metabolic pathways or thresholds of ketone availability. These findings align with previous reports indicating that ketone metabolism can modulate vascular function, inflammation, and oxidative stress factors that may impact hepatic blood flow and RBC distribution [1,4,19,23]. Further investigation is warranted to determine whether these changes are adaptive responses or potential contributors to altered liver physiology in ketone-supplemented states. Future studies should also incorporate perfusion-based controls and normalization to sinusoidal area to strengthen conclusions regarding microvascular adaptation to chronic ketone supplementation.

The observed increase in hepatic fat droplet area in the KE and MCT treatment groups suggests a formulation-dependent effect of exogenous ketones on lipid metabolism. KE and MCT both significantly elevated fat accumulation, compared to Control and KS, indicating that despite their shared capacity to elevate blood ketone levels, their metabolic consequences in the liver differ markedly, they may also contribute to increased lipid deposition in the liver under certain conditions. A previous study also reported that MCT supplemented animals had significantly larger livers, hepatomegaly, likely caused by the MCT being deposited in the liver as fat droplets [6]. In contrast, the KS group consistently showed low fat droplet counts, indicating a potential protective effect against hepatic lipid accumulation. Indeed, previous study suggested that hepatic ketogenic insufficiency is a key contributor to the initiation and progression of metabolic dysfunction-associated steatotic liver disease (MASLD) [20]. Interestingly, the combination treatment (KSMCT) moderated the lipid accumulation seen in the individual MCT and KE groups, suggesting a possible synergistic or buffering interaction. The lack of increased fat area in the KS and KSMCT groups suggests that KS formulations may exert a more lipid-neutral profile or potentially support more efficient lipid utilization. These differences could reflect variations in hepatic uptake, mitochondrial oxidation efficiency, or lipid export pathways triggered by the distinct metabolic fates of esters, salts, and MCTs. The combination of KS with MCT (KSMCT) appeared to blunt the fat-accumulating effects observed with MCT alone, which may point to a synergistic metabolic effect that warrants further mechanistic exploration. These findings underscore the importance of selecting appropriate ketone formulations for therapeutic strategies targeting liver health and metabolic disorders. These findings align with prior evidence that not all ketone supplements exert equivalent metabolic effects and highlight the importance of formulation in influencing lipid handling in hepatic tissue. Previous studies emphasize the importance of BHB concentration when used as a therapeutic, as despite its therapeutic potential, high levels of BHB may also be negative to the liver, impairing insulin signaling [19,28]. Further investigation is warranted to delineate the mechanisms by which these compounds modulate liver lipid storage and to evaluate their long-term metabolic implications.

We also explored key histological markers of hepatic health, including TNF-α and arginase expression. The observed differences in TNF-α expression among treatment groups provide important insights into the inflammatory response under different metabolic conditions. The significantly elevated TNF-α staining in the BD, KE and MCT groups suggests a pro-inflammatory effect of these interventions, possibly due to metabolic stress or hepatic stress, potentially driven by metabolic byproducts or shifts in energy substrate utilization that activate inflammatory pathways. In contrast, the KS and KSMCT groups showed markedly lower TNF-α positivity, closely resembling control animals, indicating a possible anti-inflammatory effect. This suppression may be attributed to KS-mediated modulation of immune responses or synergistic effects when combined with MCTs. The ability of KS and KSMCT to downregulate TNF-α expression may contribute to their therapeutic promise in mitigating inflammation-associated pathologies. Indeed, the anti-inflammatory effect of BHB has been described previously [29,30] for both enantiomers, D-BHB and L-BHB, while it is important to consider that L-BHB stays in the bloodstream longer [5]. Interestingly, the BD group exhibited increased TNF-α levels, suggesting that while butanediol may raise ketone levels, its impact on inflammation may differ mechanistically from KS-based interventions. These results show that ketone supplementation, particularly the KS and KSMCT groups, attenuates TNF-α expression, while BD, MCT and KE elevate inflammatory response relative to untreated controls. These findings align with previous research indicating that KSs appear to be better tolerated in chronic models [6].

The TNF-α elevation in the MCT group may be attributed to increased fatty acid oxidation or subclinical lipid-related stress. According to earlier studies, MCT oil can have mixed effects on liver inflammation. Some studies show that MCT oil can actually reduce inflammation and protect against liver damage in animal models. For instance, one study found that MCTs suppressed the expression of inflammatory cytokines and chemokines in the liver [31]. Another study indicated that MCT supplementation reduced hepatic steatosis and inflammation in obese mice [32]. However, other studies suggest that MCT oil, especially when combined with high-fructose diets, can worsen hepatic steatosis and inflammation [33]. Therefore, the impact of MCT oil on liver inflammation may depend on the specific context, including the diet, the individual’s health status, and the type of MCT oil used. It is important to note that while MCTs can be beneficial in some cases, they can also have potential drawbacks, including increased stomach upset, gastrointestinal problems, increased calorie intake and the potential for increased fat in the liver when consumed in large quantities. 

Overall, these findings highlight the nuanced roles of different ketone and lipid sources in regulating tissue inflammation, underscoring the importance of considering food intake when administering ketone formulations for therapeutic applications.

Similarly, arginase staining revealed that BD and KE supplementation showed a trend to increased hepatic arginase expression. This suggests an adaptive response to nitrogen metabolism or oxidative stress, consistent with elevated hepatic metabolic load. These findings parallel previous observations of increased hepatic enzyme activity in a case study after following the ketogenic diet [34]. Ketone electrolytes/salts, again, demonstrated a more favorable profile with minimal arginase induction, supporting their role as a liver-compatible formulation for chronic use. Together, TNF-α and arginase staining provide complementary evidence that formulation and dosage play a critical role in the hepatic safety profile of exogenous ketones.

These observations are corroborated by the H&E-stained liver morphology data (Table 1), which showed preserved hepatic structure in the control and KS groups, and increasingly disrupted morphology, fat accumulation, and RBC congestion in the BD, MCT, and KE groups. In particular, the presence of macrovesicular steatosis and vascular congestion in the KE and BD groups further supports the inflammatory and metabolic stress findings seen in TNF-α and arginase staining. Altogether, the combined histological evidence positions KSs as the most liver-compatible formulation among those tested, providing a foundation for their use in longer-term metabolic support applications.

Our results show significant group-level differences in key clinical blood biomarkers including globulin, creatinine, sodium and glucose levels, with notable distinctions between animals receiving SD, LKE, and HKE.

The observed reduction in globulin levels in the HKE group aligns with earlier reports suggesting that chronic ketone ester exposure may modulate liver protein synthesis or alter immune-related pathways [4]. Globulin, synthesized primarily in the liver, is essential for maintaining immune system functionality and protein transport [5]. A significant decrease, as seen in our HKE animals, may indicate suppressed hepatic output or altered protein metabolism. While reduced globulin has not been frequently reported in human ketone studies, Stubbs et al. [4] found modest changes in protein balance following exogenous KE ingestion, particularly at higher doses [4]. These findings raise important questions regarding long-term immune status and liver function during chronic KE use.

Elevated creatinine levels in both LKE and HKE groups suggest potential renal adaptations or stress, possibly due to the increased metabolic load imposed by ketone oxidation. While creatinine is a byproduct of muscle metabolism and may be influenced by factors, such as hydration status or protein intake, it remains a critical marker for glomerular filtration efficiency [25,35]. The fact that creatinine levels remained within physiological range is encouraging, but warrants further renal function studies in long-term human trials. Taken together, the results suggest that chronic KE supplementation can induce significant changes in clinical chemistry biomarkers. The changes in globulin and creatinine raise considerations for long-term liver and kidney monitoring.

Although histological analyses revealed formulation-dependent hepatic stress, routine blood chemistry markers were largely within reference limits. ALT, ALP, and bilirubin values did not indicate overt hepatocellular or cholestatic injury, and in fact, ALT levels trended lower in KE-treated groups compared with controls. The discrepancy highlights the importance of integrating histological, molecular, and biochemical markers to fully characterize hepatic safety, as reliance on serum enzymes alone could mask early or formulation-specific adverse effects. Although creatinine and globulin changes suggested potential renal stress in the KE groups, we did not perform complementary kidney histology or urinalysis, which would have provided direct evidence of structural or functional renal adaptations. This gap limits the depth of renal interpretation and should be addressed in future studies. Follow-up work incorporating renal histopathology and urinalysis will be critical for confirming whether the observed blood chemistry changes reflect true renal injury or adaptive responses to chronic ketone supplementation.

From a translational perspective, our findings provide insight into formulation-dependent safety profiles that are highly relevant to clinical and consumer use of exogenous ketones. Notably, the renal effects observed in the KE groups, including dose-dependent elevations in creatinine and reductions in sodium and glucose, highlight the need for careful monitoring of kidney function during prolonged KE supplementation. While creatinine remained within physiological ranges, these results underscore the importance of renal biomarkers as sensitive indicators of systemic adaptation. Together, the data suggests that formulation choice is critical: KSs appear to be better tolerated, whereas KEs and BD may warrant caution for long-term use.

No statistically significant differences were observed in albumin, ALT, ALP, BUN, potassium, chloride or bilirubin between groups, indicating that basic hepatic function largely remained stable, which further supports that chronically increasing blood BHB levels by using specific exogenous ketone supplementation may be more beneficial for protecting liver health than following a high fat diet [34]. Future studies should incorporate a broader panel of immune and renal markers, as well as histopathological assessments, to validate and contextualize these histological and biochemical changes.

Dose translation to human equivalency merits caution. While this exposure exceeds typical supplemental intakes, it was intentionally chosen to reveal potential formulation-specific adverse hepatic or renal outcomes under chronic high-dose conditions. Rodents have higher mass-specific metabolic rates, and faster clearance, and greater detox capacity than humans; therefore, simple mg/kg matching overestimates human exposure. It is also important to note that the pharmacokinetics differ by formulation and enantiomer (e.g., D- vs. L-BHB), so actual human exposures at the same HED may vary due to absorption, distribution, metabolism, and excretion. Our dose estimation is therefore approximate, intended for safety context rather than clinical recommendation. It is also important to note that rodent energy turnover and ketone clearance are faster than in humans, consequently, mg/kg matching inflates human exposure, and body surface area (BSA) conversion—while standard—still does not capture species differences in absorption, distribution, metabolism, and excretion or enantiomer kinetics. The key translational message of the outcome is formulation dependency.

Nutritional ketosis and elevated blood ketone levels have been described to have therapeutic promise not only for metabolic disorders, but also for neurological diseases, neuroregeneration, mental health conditions, and more [1,2,3,36,37]. While studies show ketone monoester (KME) did not induce any improvement in athletic performance and its acute ingestion produces mild acidosis [3,4,38], recent insights point to the significant role of another, neglected ketone molecule, the L-BHB enantiomer of ketone electrolytes/salts, not only for brain health and for reducing inflammation, but also to contribute to the greater enhancement of cardiac output [15,37]. In order to further optimize the most promising compound for chronic use, the D,L-BHB ketone electrolyte/salt formulation, dose–response relationships and the different isomers of ketone electrolyte/salts will need to be studied independently. The distinct pharmacokinetics of the D- and L-enantiomers of BHB are increasingly recognized. In the future, the contribution of D- versus L-isomers of BHB should be further elucidated, given their distinct metabolic fates and signaling capacities. While the L-isomer BHB is also bioidentical, persisting longer in circulation and potentially contributing to redox and signaling effects, has better anti-inflammatory effect and more can be found in the brain and in the muscles than the D-form after D,L-BHB supplementation, but it can’t be measured by handheld meters [5].

Even recent studies are showing that considerable variability exists among outcomes and long-term data remains limited. In a recent study, ketogenic diet (KD)-fed mice showed an increase in fat accumulation in white adipose tissue and liver after 12 weeks, which was explained by an increase in fat uptake by the liver with no changes in catabolism leading to MAFLD [36]. However, another recent review indicates that KDs significantly reduce hepatic fat content and improve metabolic parameters, including insulin sensitivity and liver enzyme levels [39] and another recent study suggests that both KD and exogenous ketone supplementation present promising strategies for managing metabolic dysfunction-associated steatotic liver disease (MASLD) [19].

Beyond effects on lipid metabolism and inflammation, BHB may also modulate cellular defense systems through activation of the Nrf2–Vitagene pathway. Nrf2 (nuclear factor erythroid 2-related factor 2) is a master regulator of antioxidant and cytoprotective genes, while the Vitagene network includes heme oxygenase-1, heat shock proteins, thioredoxin, and other stress-resistance genes. Recent studies demonstrate that BHB can increase Nrf2 nuclear translocation and upregulate Vitagene expression, thereby enhancing cellular resilience against oxidative stress [40,41,42]. This mechanism is particularly relevant in the liver, which is highly exposed to reactive oxygen species generated during fatty acid and ketone metabolism. By supporting antioxidant defenses, BHB may counterbalance some of the pro-oxidative and inflammatory challenges induced by chronic supplementation. Importantly, the potential for differential modulation by D- versus L-BHB remains to be clarified, but emerging evidence suggests that both isomers may contribute to redox regulation. Reduced inflammatory staining in KS-treated animals may, at least in part, reflect Nrf2–Vitagene activation and improved oxidative stress adaptation.

The divergent hepatic outcomes observed across formulations can be explained, at least in part, by their distinct effects on lipid handling and inflammatory signaling pathways. KEs and BD elevate circulating BHB rapidly, but their metabolism also generates acetyl-CoA surges that can overwhelm hepatic oxidative capacity, leading to diversion of acetyl-CoA toward triglyceride synthesis and lipid droplet accumulation. Importantly, BD requires oxidation by alcohol dehydrogenase (ADH), similar to ethanol metabolism, producing BHB through sequential conversion to β-hydroxybutyraldehyde and then BHB [43]. This metabolic pathway may impose additional redox stress on hepatocytes (depleting cellular NAD^+^), contributing to the pronounced steatosis and vascular congestion observed in BD-treated animals. MCTs are readily transported into mitochondria independent of carnitine and rapidly oxidized, which may explain their intermediate phenotype of ballooning hepatocytes and lipid deposition, along with modest increases in TNF-α. In contrast, KSs provide both D- and L-BHB enantiomers at lower energetic density per gram and with slower absorption kinetics, reducing acute acetyl-CoA overload and promoting more balanced lipid utilization. Furthermore, BHB itself functions as a signaling metabolite, suppressing NLRP3 inflammasome activation and downregulating pro-inflammatory cytokines, such as TNF-α, consistent with the reduced inflammatory staining seen in KS groups. Collectively, these formulation-specific differences suggest that the interplay between mitochondrial oxidation, acetyl-CoA flux, redox balance, and inflammatory cascades underlies the distinct hepatic responses observed, which may lead to better tolerability of KSs [44], while BD can depress CNS activity and even induces physical dependence, similarly to ethanol, as an earlier study suggests [45].

A limitation of this study is that only male Sprague-Dawley rats were used, which reduces potential confounding from sex-hormone variability due to sex hormone fluctuations in hepatic lipid metabolism, but limits the generalizability of the findings to both sexes. While histological, immunohistochemical, and blood chemistry analyses provided robust endpoints, additional molecular approaches in future studies, such as lipidomics or oxidative stress markers would further clarify the mechanistic pathways underlying formulation-specific effects. Behavioral or functional assessments of liver performance were not included at this time, but represent important future directions to link histological and biochemical changes with physiological outcomes. Additionally, while the chosen species doses were consistent with those previously validated [6,9] and were adjusted to approximate caloric equivalence, translation to human dosing requires careful consideration. Future work should include female cohorts and dose-ranging studies in multiple species to strengthen translational relevance. Another limitation of this study is the relatively small sample size in each treatment group. While this was sufficient to detect consistent, formulation-dependent effects across histological, immunohistochemical, and biochemical outcomes, it does reduce the ability to identify more subtle differences. Sample sizes were deliberately constrained in order to adhere to ethical guidelines that prioritize the reduction in animal use, consistent with IACUC approval and the principles of humane research. This decision reflects the ethical principle of the 3Rs (Replacement, Reduction, Refinement) in animal research. Another limitation of this study is the absence of isocaloric and mineral-matched control groups. Because KSs and KSMCT provide additional mineral loads, it is possible that some of the observed effects may reflect electrolyte shifts rather than ketone metabolism. Although all formulations were administered under standardized dosing conditions, future studies should include energy- and mineral-matched comparators (e.g., dextrose, long-chain fats, or inert mineral salts) to better isolate the specific contributions of ketones versus electrolytes. In addition, the duration of exposure (4 weeks and 83 days) is informative, but may not fully capture cumulative or delayed effects of chronic supplementation. Future studies with larger cohorts and inclusion of complementary species will help validate and extend these findings. Lastly, these changes were observed in healthy young animals, and since older animals have less robust detoxification pathways and hepatic clearance of alcohol (e.g., BD), it would be important to assess the greater potential for age-dependent toxicity and CNS effects (48).

## 4. Materials and Methods

### 4.1. Animal Model and Treatment Protocol

Animal procedures were performed in accordance with the University of South Florida Institutional Animal Care and Use Committee (IACUC) guidelines (Protocol#0006R). Male Sprague-Dawley rats (SPD, 275–325 g, Harlan Laboratories, Indianapolis, IN, USA) were housed in a temperature- and humidity-controlled environment on a 12 h light/dark cycle with free access to water and standard chow. Animals were randomly assigned to one of six treatment groups using a block randomization strategy based on starting body weight to ensure balanced distribution across conditions (n = 5 per group): H_2_O control (Control), 1,3-butanediol (BD), medium chain triglyceride (MCT), ketone ester (KE), ketone electrolyte/salt D,L-BHB (KS), and ketone electrolyte/salt + MCT mix (KSMCT, 1:1 ratio). Treatments were administered orally once daily for 4 weeks via oral gavage at a standardized volume per body weight. Caloric density of standard rodent chow, dose of ketone supplements and body weight as previously described [6,9]. Food intake and water consumption were not measured in this study.

To minimize stress associated with daily oral gavage, rats were acclimated to handling for one week prior to the start of treatment. During this period, animals were gently restrained and handled. All treatments were administered at the same time each day to reduce circadian variability. Animals were handled and restrained using identical procedures across groups, ensuring that potential stress effects were consistent and did not bias comparisons. Throughout the study, rats were monitored for signs of distress, and none were observed beyond transient responses typical of short-term handling. Although daily gavage can impose stress, our acclimation and standardized handling minimized this risk, and all groups were subjected to identical procedures, reducing the likelihood of systematic bias.

For the blood chemistry analysis, Sprague-Dawley rats were fed for 83 days with either standard rodent chow (2018 Teklad Global 18% Protein Rodent Diet (#2018), Harlan, n = 10/group) standard diet (SD)/control) or SD + ketone supplementation. Treatment groups included low-dose KE (10 g/kg b.w./day, LKE) and high-dose KE (25 g/kg b.w./day, HKE). Higher dose was used for chronic administration as previously described [9], as the rats were consuming food-integrated ketone supplementation throughout the day, not at a single time point. All experiments were approved by the University of South Florida IACUC and all efforts were made to reduce the number of animals used. Treatments were administered by personnel aware of group assignments, but immunohistochemical quantification, and serum chemistry were performed under blinded conditions. The selected doses for exogenous ketone formulations were based on previously published rodent studies [6,9], which established tolerability and metabolic efficacy of comparable ranges. Dosages were adjusted on a per-body-weight basis to ensure caloric equivalence across treatments, taking into account both the energetic contribution of the ketone formulations and the need to maintain consistency with prior protocols in Sprague-Dawley rats. This design allowed direct comparison with earlier preclinical findings while ensuring that treatment groups received similar energy loads. To facilitate translational interpretation, we estimated caloric equivalence and approximate human-equivalent doses (HEDs) by applying standard body surface area (BSA) conversion factors. Based on this estimation the doses used in this study correspond to ~0.81 g/kg/day to ~4 g/kg/day in humans. Only male Sprague-Dawley rats were used in this study to reduce potential variability associated with estrous cycle–related fluctuations in sex hormones, which are known to influence hepatic lipid metabolism and inflammatory signaling. Furthermore, the use of males was consistent with the Institutional Animal Care and Use Committee (IACUC) protocol approved for this study. We acknowledge that excluding females represents a limitation and future studies should include both sexes to determine whether these formulation-dependent effects are sexually dimorphic.

### 4.2. Liver Tissue Collection and Processing

At the end of the treatment period, animals were euthanized via deep isoflurane anesthesia and livers were harvested and fixed in 4% paraformaldehyde. Fixed tissues were then processed through graded alcohols and xylene, embedded in paraffin, and sectioned at 5 μm thickness using a microtome. Sections were mounted on glass microscope slides for histological and immunohistochemical analysis. Histological evaluations and image quantification were performed under blinded conditions. Digital images were coded prior to analysis so that investigator performing quantitative assessments of hematoxylin and eosin (H&E) staining and immunohistochemistry (TNF-α and arginase) was unaware of group assignment. This approach minimized the risk of observer bias in image scoring and quantification.

### 4.3. Hematoxylin and Eosin (H&E) Staining

H&E staining was performed following standard protocols. Tissue sections were deparaffinized in xylene and rehydrated through a graded ethanol series. Slides were stained with hematoxylin for 4–5 min to visualize cell nuclei, rinsed in tap water, and differentiated in 1% acid alcohol. After bluing in alkaline water, sections were counterstained with eosin for 1–2 min to stain cytoplasmic and extracellular components. Stained slides were dehydrated through increasing ethanol concentrations, cleared in xylene, and cover slip mounted with mounting medium. Slides were evaluated by light microscopy for changes in liver architecture, red blood cell infiltration, and lipid accumulation.

### 4.4. Histological Image Analysis

Liver tissue sections were analyzed using standardized digital image quantification methods to assess RBC area, fat droplet count, and fat droplet area. Images were captured at 40× magnification and exported in high-resolution TIFF format. Image analysis was conducted using a custom pipeline implemented with Python-based computer vision tools (Python 3. v4.x), applying consistent color space transformations and object segmentation criteria across all samples. RBCs were identified based on their characteristic red staining, and their total area within each image was calculated. Fat droplets were identified as round or near-round unstained (white) regions with sharply defined borders, consistent with intracellular lipid vacuoles. Fat droplet count was defined as the number of these lipid vacuoles per image, and fat droplet area was expressed as the cumulative cross-sectional area occupied by these droplets, normalized to total image area. Image thresholds, size filters, and shape descriptors were uniformly applied to ensure reproducibility. Data from 15 to 30 images per treatment group were collected and analyzed in a blinded manner.

### 4.5. Immunohistochemistry for TNF-α and Arginase

Immunohistochemical staining was conducted on paraffin-embedded liver sections to assess TNF-α and arginase expression. After deparaffinization and rehydration, antigen retrieval was performed using citrate buffer (pH 6.0) in water bath for 20 min. Endogenous peroxidase activity was blocked with 3% hydrogen peroxide, and non-specific binding was minimized using a protein block solution. Sections were incubated overnight at 4 °C with primary antibodies against TNF-α (1:200 dilution, Thermo Fisher Scientific, Waltham, MA, USA) or arginase (1:250 dilution, Thermo Fisher Scientific). After washing, slides were incubated with biotinylated secondary antibodies and developed using a horseradish peroxidase (HRP)-linked detection system with diaminobenzidine (DAB) as the chromogen. Sections were counterstained with hematoxylin, dehydrated, and mounted. Positive staining appeared as brown deposits, and immunoreactivity was evaluated under light microscopy.

### 4.6. Immunohistochemical Staining Quantification for TNF-α and Arginase

TNF-α and Arginase expression was assessed by quantifying the area of positive immunostaining in histological images using automated digital image analysis. High-resolution images captured at 40× magnification were analyzed using Python-based image processing workflows. First, color deconvolution was applied to isolate the DAB chromogen signal representing staining. Thresholding was performed to generate binary masks of positively stained regions, and artifacts or non-specific background were excluded using size and shape filters. The percentage of positive areas was calculated as the ratio of DAB-positive pixels to the total tissue area per image. Data from 5 animals per treatment group was collected and analyzed in a blinded manner. Group data were compiled for statistical analysis, with results expressed as mean ± standard deviation.

### 4.7. Blood Chemistry Analysis

Terminal whole blood samples (10 µL) were taken from the saphenous vein. Serum was isolated by centrifugation and stored at −80 °C until analysis. Blood chemistry parameters—including glucose, albumin, globulin, creatinine, BUN, ALT, ALP, bilirubin, sodium, potassium, and chloride—were assessed using a commercial automated biochemistry analyzer (Dri-Chem NX500, Fujifim, Tokyo, Japan) according to the manufacturer’s protocols. Data from 10 animals per group was analyzed.

### 4.8. Statistical Analysis

All statistical analyses were performed using one-way ANOVA followed by Tukey’s post hoc test to determine significance between treatment groups. A *p*-value of < 0.05 was considered statistically significant. Data are presented as mean ± standard deviation (SD) (software: GraphPad Prism version 10.4).

## 5. Conclusions

While exogenous ketones can be used as a tool to improve metabolic health and induce nutritional ketosis in addition to or without implementing a high fat, low carbohydrate diet, it is important to understand their long term impact. The results point to a selective effect of ketone supplementation on specific metabolic pathways, most notably involving liver-produced proteins and renal clearance markers. The histological findings corroborate the biochemical data, identifying ketone electrolyte salts (KSs) as the most liver-compatible formulation over chronic exposure under the experimental conditions tested.

This study demonstrates that the long-term effects of exogenous ketone supplementation on liver health and systemic biomarkers are highly dependent on the formulation used. Among the six tested compounds, ketone electrolytes/salts (KS; D,L-BHB) exhibited the most favorable safety and tolerability profiles. This formulation preserved hepatic histological architecture, elicited minimal TNF-α and arginase expression, and maintained levels closer to control values. In contrast, animals supplemented with BD and KE displayed evidence of hepatic inflammation, steatosis, and elevated markers of immune activation and metabolic stress. These findings were consistently supported by H&E staining, TNF-α, and arginase immunohistochemistry, as well as shifts in key blood biomarkers in KE group, such as decreased globulin and increased creatinine concentrations. These formulation-dependent differences underline the importance of careful consideration in the selection of exogenous ketone supplements, particularly for long-term use and in aged or health compromised subjects. While KE and BD may elevate circulating ketones significantly, their hepatic impact appears less favorable, compared to KS. The observed reductions in globulin and increases in creatinine in the KE groups also suggest potential hepatic and renal adaptations that warrant further exploration, especially in clinical settings and in patients with existing impaired liver function.

In summary, our data support the relative hepatic safety of D,L-BHB ketone electrolyte/salt (KS)-based formulations and caution against high-dose or prolonged use of certain ketone esters (KEs) and precursors (BDs, MCTs) without appropriate monitoring. Considering that exogenous ketone administration may be used clinically in people with reduced liver function (e.g., compromised or aged patients), these results need to be considered in both research and therapeutic applications. These findings contribute to a growing body of evidence needed to guide the safe and effective chronic use of exogenous ketones for metabolic, neurological diseases, as well as for health optimization.

## Figures and Tables

**Table 1:** Summary of hepatic histological features across treatment groups based on H&E staining.

Treatment	Fat Deposits	RBC Congestion	Histological Interpretation
H_2_O/Control	None	Minimal	Normal liver histology
1,3-Butanediol	Lipid accumulation	High	Steatosis and hepatic stress
MCT	Ballooning lipid vacuoles	Low	Modest hepatic stress, steatosis
Ketone Ester	Macrovesicular steatosis, fat vacuoles	Low	Pronounced steatosis and hepatic stress
Ketone Salt	None	Low	Preserved structure; best overall histological profile
MCT–Ketone Salt Mix	Microvesicular steatosis	High	Early steatosis with moderate vascular changes

**Table 2:** Summary of TNF-α staining intensity across treatment groups.

Treatment	TNF-α Expression	Interpretation
H_2_O/Control	Minimal	Baseline inflammatory state
1,3-Butanediol	High	Strong hepatic inflammatory response
MCT	High	Immune activation likely due to lipid stress
Ketone Ester	High	Elevated inflammation, likely metabolic in origin
Ketone Salt	Low	Low inflammatory signal, well tolerated
Ketone Salt + MCT Mix	Low	Low inflammation

**Table 3:** Summary of arginase staining across treatment groups.

Treatment	Arginase Expression	Interpretation
H_2_O/Control	Low	Normal hepatic metabolic activity
1,3-Butanediol	High	Elevated metabolic stress
MCT	Moderate	Moderate hepatic adaptation
Ketone Ester	High	High metabolic activity and possible stress
Ketone Salt	Low	Well tolerated with minimal hepatic activation
Ketone Salt + MCT Mix	Mild	Low hepatic activation

## Data Availability

The original contributions presented in the study are included in the article and Supplementary Materials, further inquiries can be directed to the corresponding author.

## Supplementary Materials

The following supporting information can be downloaded at: https://www.mdpi.com/article/10.3390/ph18101436/s1, Table S1: Blood chemistry markers of hepatic function that were not significantly different between SD, LKE, and HKE groups.
